# Increased liver echogenicity and liver enzymes are associated with extreme obesity, adolescent age and male gender: analysis from the German/Austrian/Swiss obesity registry APV

**DOI:** 10.1186/s12887-019-1711-4

**Published:** 2019-09-12

**Authors:** Susanne Greber-Platzer, Alexandra Thajer, Svenja Bohn, Annette Brunert, Felicitas Boerner, Wolfgang Siegfried, Andreas Artlich, Anja Moeckel, Hildegunde Waldecker-Krebs, Sophie Pauer, Reinhard W. Holl, on behalf of the APV-Study Group

**Affiliations:** 1 Division of Pediatric Pulmonology, Allergology and Endocrinology, Department of Pediatrics and Adolescent Medicine, Medical University of Vienna, Waehringer Guertel 18-20, 1090 Vienna, Austria; 2 Specialist Hospital for Pediatric Rehabilitation, 25946 Nebel, Amrum Germany; 3 Children`s Hospital Prinzessin Margaret, 64287 Darmstadt, Germany; 4 High Mountains Clinic Mittelberg, Rehabilitation for Children and Adolescents, 87466 Oy-Mittelberg, Germany; 5 Obesity Rehabilitation Center Insula, 83483 Strub, Bischofswiesen Germany; 6 Department of Paediatrics and Adolescent Medicine, Oberschwabenklinik, 88212 Ravensburg, Germany; 7 Department of Paediatrics, HELIOS Hospital of the district Gotha, 99867 Gotha, Germany; 8 Euskirchen Community Paediatric Clinic, 53879 Euskirchen, Germany; 9 Institute of Epidemiology and Medical Biometry, ZIBMT, University of Ulm, 89081 Ulm, Germany

**Keywords:** Childhood obesity, Non-alcoholic fatty liver disease, Male gender, Liver echogenicity, Liver enzymes, Impaired glucose tolerance

## Abstract

**Background:**

Childhood obesity is often associated with non-alcoholic fatty liver disease (NAFLD), the most common chronic liver disease in pediatrics.

**Methods:**

This multi-center study analyzed liver echogenicity and liver enzymes in relation to obesity, age, gender and comorbidities. Data were collected using a standardized documentation software (APV) from 1.033 pediatric patients (age: 4–18 years, body mass index = BMI: 28–36 kg/m^2^, 50% boys) with overweight (BMI >90th percentile), obesity (BMI >97th percentile) or extreme obesity (BMI > 99.5th percentile) and obesity related comorbidities, especially NAFLD from 26 centers of Germany, Austria and Switzerland. Liver enzymes aspartate aminotransferase (AST), alanine-aminotransferase (ALT) and gamma glutamyltransferase (gammaGT) were evaluated using 2 cut-off values a) > 25 U/L and b) > 50 U/L. Multiple logistic regression models were used for statistical analysis.

**Results:**

In total, 44% of the patients showed increased liver echogenicity. Liver enzymes > 25 U/L were present in 64% and > 50 U/L in 17%. Increased liver echogenicity was associated with elevated liver enzymes (> 25 U/L: odds ratio (OR) = 1.4, 95% CI: 1.1–1.9, *P* < 0.02; > 50 U/L: OR = 3.5, 95% CI: 2.4–5.1, *P* < 0.0001). Extreme obesity, adolescence and male gender were associated with increased liver echogenicity (extreme obesity vs overweight OR = 3.5, 95% CI: 1.9–6.1, *P* < 0.0001; age > 14 years vs age < 9 years OR = 2.2, 95% CI: 1.4–3.5, *P* < 0.001; boys vs girls OR = 1.6, 95% CI: 1.2–2.0, *P* < 0.001) and elevated liver enzymes (extreme obesity vs overweight > 25 U/L: OR = 4.1, 95% CI: 2.4–6.9, *P* < 0.0001; > 50 U/L: OR = 18.5, 95% CI: 2.5–135, *P* < 0.0001; age > 14 years vs age < 9 years > 50 U/L: OR = 1.9, 95% CI: 1.0–3.7, *P* > 0.05; boys vs girls > 25 U/L: OR = 3.1, 95% CI: 2.4–4.1, *P* < 0.0001; > 50 U/L: OR = 2.1, 95% CI: 1.5–2.9, *P* < 0.0001). Impaired glucose metabolism showed a significant correlation with elevated liver enzymes > 50 U/L (OR = 4.4, 95% CI: 1.6–11.8, *P* < 0.005). Arterial hypertension seemed to occur in patients with elevated liver enzymes > 25 U/L (OR 1.6, 95% CI: 1.2–2.0, *P* < 0.005).

**Conclusions:**

NAFLD is strongly related to extreme obesity in male adolescents. Moreover impaired glucose tolerance was observed in patients with elevated liver enzymes > 50 U/L, but arterial hypertension was only present in patients with moderately elevated liver enzymes > 25 U/L.

## Background

Childhood obesity has increased worldwide over the past three decades [1, 2]. According to Kromeyer-Hauschild, overweight in children and adolescents is defined as a body mass index (BMI) >90th percentile, obesity as BMI >97th percentile and extreme obesity as BMI > 99.5th percentile [3]. As a result of the increasing prevalence of obesity in childhood, especially in industrialized countries, non-alcoholic fatty liver disease (NAFLD) has become the most common cause of chronic liver disease in the pediatric age group [1, 4].

NAFLD is defined as a spectrum of fatty liver disease that occurs in the absence of primary liver diseases or secondary causes of hepatic fat accumulation such as excessive alcohol consumption, drug use, hepatitis C infection, or hereditary disorders (e.g. Wilson’s disease, haemochromatosis) [5]. NAFLD can be histologically subclassified into the non-alcoholic fatty liver (NAFL) and non-alcoholic steatohepatitis (NASH) [6]. Excessive hepatic accumulation of triglycerides and free fatty acids is suggested as a cause of NAFLD. This accumulation initiates the production of toxic reactive oxygen species, the release of inflammatory cytokines, and leads to mitochondrial dysfunction, which results in steatohepatitis and progression to liver fibrosis [7].

Insulin resistance seems to favor the influx of free fatty acids into hepatocytes, which increases the triglyceride content in the liver. Hence, impaired glucose tolerance is a significant risk factor for hepatic steatosis [8, 9].

The gold standard for the diagnosis of NAFLD is liver biopsy, which is not feasible in most children and adolescents [10]. Because of an increased risk of complications, the current guidelines recommend non-invasive markers like aminotransferase levels and imaging techniques like liver echogenicity in children and adolescents to verify the diagnosis of fatty liver disease [11, 6]. Abdominal ultrasonography is the most widely used imaging method in NAFLD and sensitivity ranges from 60 to 94%, and specificity from 84 to 100% [11]. Sonographic features of steatosis hepatis include hepatomegaly with smooth liver surface, blunted liver edge, increased echogenicity, mostly normal parenchymal echotexture, decreased acoustic penetration, rarefication of liver veins, and loss of the echogenic borders of portal vessels [12].

Magnetic resonance imaging and spectroscopy show the highest sensitivity and specificity for the detection of hepatic fat content, but is time-consuming and expensive, and therefore not suitable for routine diagnostics [11]. To establish diagnosis of NAFLD and to screen children for possible obesity-related metabolic complications, triglycerides, cholesterol, basal and/or stimulated glucose and/or insulin, and liver enzymes like aspartate aminotransferase (AST), alanine-aminotransferase (ALT) and gamma glutamyltransferase (gammaGT) are measured [11, 6]. For NAFLD screening in obese pediatric patients, no reference values for liver enzymes are available. However, cut-off levels for elevated liver enzymes of > 25 U/L [13] as well as > 50 U/L [14] have been published.

There is no proven specific therapy for the spectrum of non-alcoholic fatty liver disease. Most therapeutic approaches focus on lifestyle interventions, especially physical activities and diet [15].

NAFLD is associated with a higher risk of hepatic and non-hepatic complications and mortality [16, 17]. Insulin resistance and visceral obesity are frequently associated with NAFLD [18]. The rate of NAFLD progression from benign steatosis to non-alcoholic steatosis hepatis is unknown. However, elevated gammaGT seems to be a clear predictor for liver fibrosis [19].

Therefore, clinical diagnosis of NAFLD in pediatric patients should be based on the presence of ≥1 features of the metabolic syndrome (usually in children > 10 years), abdominal ultrasound showing liver brightness, and increased transaminase activity. Other steatotic and non-steatotic diseases should be excluded [20].

This multi-center study aimed to determine the correlation between NAFLD measured by liver echogenicity and/or liver enzymes and comorbidities like hypertension, dyslipidemia and impaired glucose metabolism in obese children and adolescents.

## Methods

### Study cohort

Based on the German Guidelines for the Diagnosis and Treatment of Overweight Children and Adolescents (www.a-g-a.de), a computer software using the visual FoxPro 9.0 compiler was developed in 1999 (Adipositas-Patienten-Verlaufsdokumentation; APV) for the standardized prospective documentation of children and adolescents receiving specialized obesity care (www.a-p-v.de). Anthropometric and metabolic parameters are documented longitudinally at each participating center with the aid of this software. In total, 26 centers specialized in pediatric obesity from Germany, Austria and Switzerland participated in this study.

Our study included all patients aged four to 18 years with a BMI >90th percentile, with available data for liver sonography and levels of serum liver enzymes (AST/ ALT/gammaGT) over the time period from 1st January 2005 to 31st December 2014.

Patients suffering from primary genetic defects associated with obesity (e.g. leptin and leptin receptor deficiency, proopiomelanocortin deficiency, melanocortin receptor 4 deficiency, prohormone convertase deficiency, brain-derived neurotrophic factor and tropomyosin receptor kinase B insufficiency, single-minded transcription factor-1 insufficiency), endocrinological disorders (e.g. hypothyroidism, Cushing disease, growth hormone deficiency, hypothalamic obesity, hypogonadism, insulinoma, pseudohypoparathyroidism), and other obesity related syndromes (e.g. Prader-Willi syndrome, Bardet–Biedl syndrome, Beckwith–Wiedemann syndrome, Alström–Hallgren syndrome, Carpenter syndrome, Cohen syndrome), or secondary obesity and chronic alcohol consumption (≥ 20 g/d) were excluded from the study.

### Definitions

- Overweight was defined as BMI >90th percentile, obesity was defined as BMI >97th percentile and extreme obesity as BMI > 99.5th percentile according to Kromeyer-Hauschild [3].
- Levels of serum liver enzymes (AST/ALT/gammaGT) were considered as elevated according to two different cut-off levels; a) > 25 U/L [13] and b) > 50 U/L for one parameter [14, 21].
- Impaired glucose metabolism (IGM) was defined as fasting glucose > 100 mg/dl, two-hour blood glucose > 140 mg/dl in the oral glucose tolerance test (OGTT), or glycated hemoglobin (HbA1C) > 5.7% [22].
- Hypertension in children and adolescents was defined as age and gender adjusted systolic blood pressure (BP), and/or diastolic BP >95th percentile [23].
- Dyslipidemia was defined as hypercholesterolemia for low-density lipoprotein cholesterol (LDL-C) > 130 mg/dl or total cholesterol (TC) > 190 mg/dl, high density lipoprotein cholesterol (HDL-C) < 35 mg/dl, hypertriglyceridemia with triglycerides (TG) > 150 mg/dl, or combined hyperlipidemia consisting of LDL-C > 130 mg/dl + TG > 150 mg/dl [23].

### Statistical analysis

Data are presented as mean ± standard deviation (±SD) for normally distributed outcomes and median (first to third quartile) for outcomes with skewed distribution. Nominal data are shown as number (n) and percentage (%). Wilcoxon rank sum test was used to compare continuous variables, χ2-test was used for binomial data. Correlations were tested by Spearman’s test. As multiple comparisons were performed, *p*-values were adjusted using the Bonferroni step-down correction (Holm method 21). A probability value of < 0.05 was considered significant. The results are based on multivariable logistic regression analysis.

Multiple logistic regression analysis was used to identify covariates affecting the dependent variables liver enzymes or liver echogenicity, described by odds ratio (OR) point estimates with their 95% Wald confidence interval (CI) limits. The independent variables included in the analysis were age groups, gender, BMI categories, hypertension, dyslipidemia and abnormal carbohydrates metabolism.

## Results

In total, 1.033 patients, 519 (50.2%) female and 514 (49.8%) male children and adolescents, from 26 centers in Germany, Switzerland and Austria were included in the study. The mean age of the patients was 13 years (median = 13.4; lower quartile = 11.2 / upper quartile = 15.2).

Pubertal development on female breast scale was examined in 319 (61%) girls and the majority (33%) was in Tanner stage 5. Pubertal development on male external genital scale was assessed in 296 (58%) boys and the majority (28%) showed Tanner stage 2. Pubic hair scale was evaluated in 648 (63%) of all patients. The majority ofthe girls (31%) showed Tanner stage 5, while the majority of the boys (24%) was in Tanner stage 2. The patients were divided into three age groups. Group 1 included all children aged between 4 and 8.9 years. This group consisted of 101 (10%) patients. Group 2 included all children aged between 9 and 13.9 years and consisted of 512 (49%) patients. Group 3 included all adolescents aged between 14 and 18 years and consisted of 420 (41%) patients.

The mean BMI was 32 kg/m^2^ (median = 32.02; [27.97 / 35.82]), mean waist circumference was 96 cm (median = 100; [89 / 112]), mean hip circumference was 106 cm (median = 109; [96 / 119]), mean waist-to-hip ratio was 0.946 (median = 0.953; [0.891 / 1.002]).

In total, 74 (7%) patients were defined as overweight, 340 (33%) as obese and 619 (60%) as extreme obese.

Liver echogenicity was normal in 575 (56%) participants and increased in 458 (44%) participants.

Using the cut-off for liver enzymes > 25 U/L (AST/ALT/gammaGT), 369 (36%) participants had normal levels with a mean value of 14 U/L for AST, 12 U/L for ALT and 14 U/L for gammaGT. Liver enzymes were elevated in 664 (64%) patients with a mean value of 36 U/L for AST, 43 U/L for ALT and 26 U/L for gammaGT. Using the cut-off for liver enzymes > 50 U/L, AST/ALT/gammaGT were normal in 859 (83%) participants with a mean value of 23 U/L for AST, 22 U/L for ALT and 18 U/L for gammaGT. Elevated levels were found in 174 (17%) patients with a mean value of 54 U/L for AST, 79 U/L for ALT and 44 U/L for gammaGT. For both groups (liver enzymes > 25 U/L or > 50 U/L) the highest pathological liver enzyme values were found for ALT.

Among the patients, 178 (18%) were categorized with hypercholesterolemia with a mean value of 137 mg/dL for LDL-C, 209 mg/dL for TC and 52 mg/dL for HDL-C (including 5.8% with HDL-C < 35 mg/dL), 131 (13%) with hypertriglyceridemia with a mean value of 219 mg/dL triglycerides, and 64 (6%) with combined hyperlipidemia with a mean value of 134 mg/dL for LDL-C, and 218 mg/dL for TG. 475 (47%) patients showed lipid values in the normal range. Impaired glucose metabolism could be detected in 21 (2%) cases with a mean value of 115 mg/dL for fasting glucose, and/or 167 mg/dL for 2-h OGTT levels, and/or a mean HbA1C level of 5.9%. 420 (43%) patients were classified as hypertensive.

Mean values of liver enzymes were 25 U/L for AST, 25 U/L for ALT and 20 U/L for gammaGT in patients with normal liver echogenicity and 33 U/L for AST, 40 U/L for ALT and 27 U/L for gammaGT in patients with increased liver echogenicity. In multivariable analysis (logistic regression model), elevated liver enzymes were relevant covariates for the presence of increased liver echogenicity. Elevated liver enzymes with a cut-off > 25 U/L predicted increased liver echogenicity (OR = 1.41, 95% CI: 1.1–1.9, *P* < 0.02). Based on the cut-off > 50 U/L the OR for increased liver echogenicity was 3.55, 95% CI: 2.4–5.1, *P* < 0.0001.

Relevant covariates for the presence of increased liver echogenicity were extreme obesity, male gender and adolescent age. Extreme obesity was strongly associated with increased liver echogenicity (OR = 3.45, 95% CI: 1.9–6.1, *P* < 0.0001). Increased liver echogenicity was more prevalent in boys than in girls (OR = 1.57, 95% CI: 1.2–2.0, *P* = 0.0006). An age older than 14 years predicted increased liver echogenicity (OR = 2.21, 95% CI: 1.4–3.5, *P* = 0.0007), compared to an age younger than 9 years. Results are presented in Table 1.

**Table 1:** OR for the prediction of increased liver echogenity [95% CI]

	OR [95% CI]
Liver enzymes > 25 U/L	1.41 [1.1–1.9]
Liver enzymes > 50 U/L	3.55 [2.4–5.1]
Extreme obesity	3.45 [1.9–6.1]
Male gender	1.57 [1.2–2.0]
Age > 14 years	2.21 [1.4–3.5]

Results are based on multivariable logistic regression analysis

The elevation of liver enzymes was also associated with obesity, male gender and older age.

Elevation of liver enzymes > 25 U/L was correlated with extreme obesity (OR = 4.1, 95% CI: 2.4–6.9, *P* < 0.0001 for extremely obese children compared to obese children) and male sex (OR = 3.1, 95% CI: 2.4–4.1, *P* < 0.0001 for boys in relation to girls).

Elevation of liver enzymes > 50 U/L correlated with obesity (OR = 9.33, 95% CI: [1.3–69.1], *P* = 0.0589 compared to overweight). This association was significant in extremely obese children (OR = 18.5, 95% CI: [2.5–135], *P* < 0.0001 *P* = 0.001 compared to obese children). Elevated liver enzymes were more frequently seen in boys than in girls (OR = 2.1, 95% CI: 1.5–2.9, *P* < 0.0001 for boys in relation to girls). An age older than 14 years predicted elevated serum aminotransferase levels (OR = 1.93, 95% CI: 1.0–3.7, P = 0.0589 compared to an age younger than 9 years).

We found a positive association between arterial hypertension and elevated liver enzymes > 25 U/L compared to normal liver enzymes (OR = 1.6, 95% CI: 1.2–2.1, *P* = 0.0026). However, this interaction was not demonstrated for liver echogenicity nor for liver enzymes > 50 U/L. For other obesity related comorbidities, such as impaired glucose metabolism or dyslipidemia, we could not detect an effect on liver echogenicity nor on elevated liver enzymes > 25 U/L. A positive association was only detected between impaired glucose metabolism and elevated liver enzymes > 50 U/L (OR = 4.4, 95% CI: 1.6–11.8, *P* = 0.0032). Details are shown in Table 2.

**Table 2:** Normal and pathological liver echogenicity / normal and elevated serum liver enzymes. Results are based on multivariable logistic regression analysis

		Normal liver echogenicity	Pathological liver echogenicity	Normal liver enzymes (< 25 U/L)	Elevated liver Enzymes (> 25 U/L)	Normal liver enzymes (< 50 U/L)	Elevated liver Enzymes (> 50 U/L)
n (%)		*n* = 575 (56%)	*n* = 458 (44%)	*n* = 369 (36%)	n = 664 (64%)	*n* = 859 (83%)	*n* = 174 (17%)
Gender	female n (%)	310 (54%)	209 (46%)	123 (33%)	391 (59%)	454 (53%)	65 (37%)
	male n (%)	265 (46%)	249 (54%)	246 (67%)	273 (41%)	405 (47%)	109 (63%)
Age	4–8.9 years n (%)	68 (12%)	33 (7%)	36 (10%)	65 (10%)	89 (10%)	12 (7%)
	9–13.9 years n (%)	309 (54%)	203 (44%)	192 (52%)	320 (48%)	438 (51%)	74 (42%)
	14–18 years n (%)	198 (34%)	222 (49%)	141 (38%)	279 (42%)	332 (39%)	88 (51%)
BMI	90.-97. percentile n (%)	57 (10%)	17 (4%)	46 (13%)	28 (4%)	73 (8%)	1 (1%)
	97.-99.5. percentile n (%)	226 (39%)	114 (25%)	134 (36%)	206 (31%)	298 (35%)	42 (24%)
	> 99.5. percentile n (%)	292 (51%)	327 (71%)	189 (51%)	430 (65%)	488 (57%)	131 (75%)
Obesity related Co-morbidities	IGM n (%)	11 (2%)	10 (2%)	5 (1%)	16 (3%)	12 (2%)	9 (6%)
	dyslipidemia n (%)	27 (5%)	36 (8%)	16 (4%)	47 (7%)	49 (6%)	14 (8%)
	arterial hypertension n (%)	227 (41%)	193 (45%)	123 (35%)	297 (47%)	343 (42%)	77 (47%)
Tanner stage	Breast for girls n (%)	173 (55%)	146 (45%)	156 (49%)	163 (51%)	277 (87%)	42 (13%)
	Tanner stage 0 n (%)	3 (2%)	1 (1%)	1 (1%)	3 (2%)	4 (2%)	0 (0%)
	Tanner stage 1 n (%)	34 (20%)	22 (15%)	32 (20%)	24 (15%)	51 (18%)	5 (12%)
	Tanner stage 2 n (%)	25 (14%)	19 (13%)	22 (14%)	22 (13%)	38 (14%)	6 (14%)
	Tanner stage 3 n (%)	35 (20%)	17 (12%)	25 (16%)	27 (17%)	47 (17%)	5 (12%)
	Tanner stage 4 n (%)	28 (16%)	31 (21%)	25 (16%)	34 (21%)	48 (17%)	11 (26%)
	Tanner stage 5 n (%)	48 (28%)	56 (38%)	51 (33%)	53 (32%)	89 (32%)	15 (36%)
Tanner stage	Genitalia for boys n (%)	144 (49%)	152 (51%)	71 (24%)	225 (76%)	236 (80%)	60 (20%)
	Tanner stage 0 n (%)	1 (1%)	0 (0%)	0 (0%)	1 (0%)	1 (1%)	0 (0%)
	Tanner stage 1 n (%)	33 (23%)	37 (24%)	24 (34%)	46 (20%)	59 (25%)	11 (18%)
	Tanner stage 2 n (%)	40 (28%)	43 (28%)	25 (35%)	58 (26%)	65 (27%)	18 (30%)
	Tanner stage 3 n (%)	36 (25%)	25 (17%)	12 (17%)	49 (22%)	50 (21%)	11 (18%)
	Tanner stage 4 n (%)	27 (18%)	39 (26%)	8 (11%)	58 (26%)	54 (23%)	12 (20%)
	Tanner stage 5 n (%)	7 (5%)	8 (5%)	2 (3%)	13 (6%)	7 (3%)	8 (14%)
Tanner stage	Pubic hair girls n (%)	193 (56%)	153 (44%)	167 (48%)	179 (52%)	302 (87%)	44 (13%)
	Tanner stage 0 n (%)	4 (2%)	3 (2%)	2 (1%)	5 (3%)	7 (2%)	0 (0%)
	Tanner stage 1 n (%)	41 (21%)	26 (17%)	37 (22%)	30 (17%)	61 (20%)	6 (14%)
	Tanner stage 2 n (%)	32 (17%)	16 (11%)	21 (13%)	27 (15%)	39 (13%)	9 (20%)
	Tanner stage 3 n (%)	34 (18%)	15 (10%)	28 (17%)	21 (12%)	47 (15%)	2 (5%)
	Tanner stage 4 n (%)	31 (16%)	31 (20%)	27 (16%)	35 (19%)	50 (17%)	12 (27%)
	Tanner stage 5 n (%)	48 (25%)	60 (39%)	50 (30%)	58 (32%)	93 (31%)	15 (34%)
	Tanner stage 6 n (%)	3 (1%)	2 (1%)	2 (1%)	3 (2%)	5 (2%)	0 (0%)
Tanner stage	Pubic hair boys n (%)	157 (52%)	145 (48%)	62 (21%)	240 (79%)	234 (77%)	68 (23%)
	Tanner stage 0 n (%)	1 (1%)	1 (1%)	0 (0%)	2 (1%)	2 (1%)	0 (0%)
	Tanner stage 1 n (%)	39 (25%)	42 (29%)	22 (35%)	59 (24%)	65 (28%)	16 (24%)
	Tanner stage 2 n (%)	40 (25%)	31 (21%)	23 (37%)	48 (20%)	56 (24%)	15 (22%)
	Tanner stage 3 n (%)	38 (24%)	20 (14%)	9 (15%)	49 (20%)	47 (20%)	11 (16%)
	Tanner stage 4 n (%)	28 (18%)	39 (27%)	5 (8%)	62 (26%)	54 (23%)	13 (19%)
	Tanner stage 5 n (%)	10 (6%)	12 (8%)	3 (5%)	19 (8%)	10 (4%)	12 (18%)
	Tanner stage 6 n (%)	1 (1%)	0 (0%)	0 (0%)	1 (1%)	0 (0%)	1 (1%)

n (number), percent (%), *BMI* Body Mass Index, *IGM* Impaired Glucose Metabolism

Table 3 shows the comparison of liver echogenicity and elevated liver enzymes with the cut-off levels > 25 U/L and > 50 U/L. All three liver enzymes (AST, ALT and gammaGT) significantly differed between the groups (*P* < 0.0001) (Table 3).

**Table 3:** Comparison with Wilcoxon rank sum test and using Bonferroni step-down correction for *p*-values

n (%)	Normal liver echogenicity	Pathological liver echogenicity	*P*-value	Normal liver enzymes (< 25 U/L)	Elevated liver Enzymes (> 25 U/L)	*P*-value	Normal liver enzymes (< 50 U/L)	Elevated liver Enzymes (> 50 U/L)	*P*-value
	*n* = 575 (56%)	*n* = 458 (44%)		*n* = 369 (36%)	*n* = 664 (64%)		*n* = 859 (83%)	*n* = 174 (17%)	
	Mean ± SD			Mean ± SD			Mean ± SD		
Age (years)	12.7 ± 2.9	13.5 ± 2.8	< 0.0001	12.9 ± 2.9	13.1 ± 2.8	n.s.	12.9 ± 2.9	13.8 ± 2.8	0.001
Height (cm)	157.9 ± 14.6	161.6 ± 13.5	n.s.	158.3 ± 14.2	160.2 ± 14.3	n.s.	158.7 ± 14.3	163.9 ± 13.6	n.s.
Weight (kg)	79.4 ± 24.6	90.3 ± 25.5	< 0.0001	79.5 ± 24.4	86.8 ± 25.9	0.003	82.0 ± 24.8	95.3 ± 26.5	< 0.0001
BMI (kg/m^2^)	31.2 ± 5.9	34.0 ± 6.2	< 0.0001	31.1 ± 6.0	33.2 ± 6.1	< 0.0001	31.9 ± 6.1	34.9 ± 5.9	< 0.0001
AST (U/L)	25.0 ± 15.7	33.3 ± 27.4	< 0.0001	14.4 ± 9.3	35.8 ± 23.1	< 0.0001	23.2 ± 10.4	54.5 ± 38.4	< 0.0001
ALT (U/L)	24.9 ± 23.0	40.1 ± 37.0	< 0.0001	11.9 ± 9.0	42.6 ± 33.3	< 0.0001	22.1 ± 12.3	78.5 ± 47.7	< 0.0001
gammaGT (U/L)	19.7 ± 17.9	27.3 ± 22.9	< 0.0001	14.2 ± 5.2	26.2 ± 23.1	< 0.0001	18.2 ± 7.1	43.7 ± 38.6	< 0.0001
SBP (mmHg)	123.9 ± 14.9	126.3 ± 14.2	n.s.	122.5 ± 15.6	126.3 ± 13.9	0.0002	124.3 ± 14.9	128.1 ± 13.1	0.016
DBP (mmHg)	73.2 ± 10.3	74.3 ± 10.5	n.s.	72.9 ± 10.3	74.1 ± 10.4	n.s.	73.3 ± 10.4	75.8 ± 10.2	0.031
TC (mg/dL)	128.2 ± 72.1	126.0 ± 76.7	n.s.	93.2 ± 81.7	146.5 ± 61.7	< 0.0001	123.8 ± 75.2	144.3 ± 66.1	n.s.
HDL-C (mg/dL)	39.5 ± 35.1	33.0 ± 21.5	< 0.0001	27.7 ± 32.8	41.7 ± 27.0	< 0.0001	36.7 ± 32.1	36.2 ± 16.4	n.s.
LDL-C (mg/dL)	76.5 ± 46.5	76.2 ± 50.9	n.s.	54.9 ± 50.3	88.7 ± 42.8	< 0.0001	73.7 ± 47.9	90.2 ± 49.2	0.016
TG (mg/dL)	78.1 ± 78.0	88.4 ± 73.0	0.014	61.9 ± 77.9	94.5 ± 72.2	< 0.0001	78.6 ± 76.7	102.6 ± 68.8	< 0.0001
Fasting glucose (mg/dL)	69.9 ± 32.6	77.6 ± 50.8	n.s.	52.6 ± 40.0	84.8 ± 38.3	< 0.0001	71.2 ± 44.4	84.4 ± 21.0	0.0002
OGTT (mg/dL)	87.9 ± 43.8	96.5 ± 42.6	n.s.	66.4 ± 52.6	106.6 ± 27.6	< 0.0001	88.2 ± 43.9	110.0 ± 35.9	0.0006
HbA1C (%)	5.3 ± 0.5	5.7 ± 6.1	n.s.	6.1 ± 7.8	5.3 ± 0.4	n.s.	5.6 ± 4.8	5.3 ± 0.6	n.s.

n (number), *ALT* Alanine-Aminotransferase, *AST* Aspartat-Aminotransferase, *BMI* Body Mass Index, *DBP* Diastolic Blood Pressure, *gammaGT* gamma Glutamyltransferase, *HbA1C* Glycated Haemoglobin, *HDL-C* High Density Lipoprotein Cholesterol, *LDL-C* Low-Density Lipoprotein Cholesterol, *OGTT* Oral Glucose Tolerance Test, *SBP* Systolic Blood Pressure, *TC* Total Cholesterol, *TG* Triglycerides, *SD* Standard Deviation

## Discussion

The main findings of this study are a strong association of increased BMI, adolescent age and male sex with elevated liver echogenicity and liver enzymes. With respect to comorbidities, only arterial hypertension was associated with impaired glucose metabolism and elevated liver enzymes.

The NAFLD prevalence was reported as 83% in extremely obese adolescents [24]. In the current study, pathological liver echogenicity was present in 44% (*n* = 458) of the patients in our study population. 71% (*n* = 327) of the patients in our extremely obese group were affected by increased liver echogenicity. Elevated liver enzymes > 25 U/L were found in 64% (*n* = 664) of all children and adolescents. Out of these patients, the extremely obese group accounted for 65% (*n* = 439) of the cases. Elevated liver enzymes > 50 U/L were detected in 17% (*n* = 174) in our study population. In the extremely obese group, increased liver enzymes > 50 U/L were observed in 75% (*n* = 131). Fat infiltration must affect > 20% of hepatocytes to be visualized by liver ultrasound [25]. In NAFLD, literature reports a strong correlation between liver echogenicity and liver biopsy. Therefore, ultrasonography can be postulated as an easily, available, feasible and safe screening method [26].

For pediatric NAFLD screening, liver enzymes are surrogate markers. In childhood, reference levels for ALT and AST are gender independent but slightly increase from preschool to prepubertal age. From puberty onwards, ALT and AST levels are higher in boys. The upper limit of the ALT/AST normal range is currently set at 25–35 U/L depending on the literature [27, 28] [29]. The optimal cut-off level is not known for the specific pediatric population. A higher cut-off level for liver enzymes increases the specificity but decreases the sensitivity. Therefore, within this study, two different cut-off levels for liver enzymes were used. Wiegand et al. analyzed serum aminotransferase levels in a cohort of 16.390 overweight and obese children and adolescents in a multi-center APV study and showed results similar to our current data. They demonstrated that elevated levels of ALT and/or AST > 50 U/L were present in 11% of patients, predominantly in boys (boys vs girls; 14.4:7.4%; *P* < 0.001), extreme obesity (obese vs extremely obese; 9.5:17.0%; *P* < 0.001) and adolescent age (< 12 vs > 12 years; 8:12%; *P* < 0.001; adjusted for BMI). Moreover, ALT > 50 U/L significantly correlated with increased fasting insulin and higher BMI-SDS [14]. In a further multi-center APV study, Wiegand et al. examined the association of gamma-glutamyl transferase to BMI, sex, and age in 68.415 children. In this study, gammaGT > 50 U/L was strongly associated with extreme obesity (OR = 27.13, 95% CI: 15.07–48.85) and male sex (OR = 2.60, 95% CI: 2.03–3.31) [21]. However, gammaGT is a sensitive but non-specific biomarker for NAFLD [30].

The relationship of elevated liver enzymes and increased liver echogenicity with male sex and adolescent age (≥Tanner stage 2) might be explained by a pubertal increase of androgens affecting the pathogenesis of NAFLD in obesity. The role of androgen in the pathogenesis of NAFLD has been investigated by Dai et al. Androgen receptor (AR) signaling plays an important role in the development and progression of several liver diseases including NAFLD [31].

The role of estrogen in the pathogenesis of NAFLD is described as protective. In a randomized placebo-controlled trial in women with type 2 diabetes mellitus, hormone replacement therapy improved liver function tests compared to placebo [32]. In a mouse-model, it was shown that knock out mice for estrogen receptor alpha showed higher - microvascular steatosis and ALT levels if fed with high fat diet compared to wild type animals [33].

Fraser et al. found that elevated ALT levels were more frequent in male adolescents (12,4%) than in female ones (3,5%). Elevated ALT levels were strongly linked to elder age and higher fasting insulin levels [34].

A possible explanation for the association between adolescent age and elevation of liver enzymes is that insulin sensitivity decreases during puberty [35]. Insulin resistance plays an important role in the pathogenesis of NAFLD. Insulin resistance favors the influx of fatty acids into hepatocytes. This leads to accumulation of intrahepatic triglycerides and might result in hepatic steatosis [9].

This interaction between NAFLD and impaired glucose metabolism was observed by means of ultrasonography in a study of 169 obese adolescents. The prevalence of NAFLD was significantly higher in pubertal children (61.9%) than in pre-pubertal children (40.8%), whereas homeostasis model assessment-insulin resistance (HOMA-IR) values were elevated in both groups. The study described a positive correlation between the liver ultrasound score and HOMA-IR in pubertal children [36].

Jun-Fen Fu et al. analyzed the correlation of NAFLD and metabolic syndrome in a cohort of 861 obese children. The prevalence of metabolic syndrome was much higher in children with NAFLD than in children with normal liver function. Based on ultrasound scales, the presence of moderate and severe liver fatty infiltration carried a high risk for hypertension, dyslipidemia and impaired fasting glucose [37].

In our study, there was a strong association between impaired glucose metabolism and elevated liver enzymes > 50 U/L (*P* < 0.005), but none between impaired glucose metabolism and liver echogenicity. Moreover, we did not detect an association between elevated liver enzymes > 50 U/L or liver echogenicity and other obesity related comorbidities like dyslipidemia or arterial hypertension. However, when using the lower cut-off level for liver enzymes (> 25 U/L), a correlation with arterial hypertension could be noticed (*P* < 0.005).

Our retrospective analysis enrolled children and adolescents with obesity related comorbidities. Only 2% (*n* = 21) of the patients suffered from impaired glucose metabolism and 6% (*n* = 64) from dyslipidemia. Other known risk factors for the development of NAFLD were found more frequently: 18% of the patients showed hypercholesterolemia, 13% had hypertriglyceridemia, and arterial hypertension was present in 43% of the patients. However, no statistically significant associations between obesity related comorbidities and NAFLD were detected.

Our study indicates a high prevalence of liver disease with increasing obesity in children and adolescents, but a lower prevalence of comorbidities like dyslipidemia or impaired glucose metabolism. It is well known that excessive ectopic fat deposition in liver is causative for NAFLD. There is evidence that this hepatic inflammation is a causative factor in the development of hepatic and systemic insulin resistance. This concept was demonstrated by D’Adamo et al., who assessed hepatic fat content in obese adolescents with NAFLD. They compared insulin sensitivity in 23 obese adolescents with high hepatic fat fraction (HFF > 5.5%) to 20 obese adolescents with low HFF (HFF < 5.5%), matched for age, Tanner stage, BMI, percentages of body fat, intraabdominal and intramyocellular lipid content. The intrahepatic fat content was associated with impaired insulin resistance and ß-cell dysfunction [38].

The effects of steatohepatitis on insulin resistance have been elucidated in a study by Cali et al. including 118 obese adolescents. In this trial, increasing severity of fatty liver was associated with a pro-inflammatory milieu, independent of total body fat. Thus, mild to moderate hepatic steatosis was associated with an imbalance between anti- and pro-inflammatory markers which might induce inflammation in the liver. This inflammation could be causative for insulin signaling disorders in adolescents, clinically presenting as insulin resistance, glucose intolerance and type 2 diabetes mellitus [39]. It seems obviously that NAFLD is linked to the development of type 2 diabetes mellitus. In adults with NAFLD followed up over a period of 14 years, Ekstedt et al. could show an increase in the prevalence of impaired glucose tolerance or type 2 diabetes from 9% at baseline to 78% at the end of the observational period [40].

A limitation of our study is that liver biopsy was not used for NAFLD diagnosis. However, biopsy is invasive, expensive and not feasible for our young patient group [41]. Conversely, abdominal ultrasonography is characterized by safety, validity, and cost-effectiveness. Therefore, it is an easily accessible screening tool for children and adolescents [42]. However, the assessment of liver echogenicity by ultrasonography comparing liver brightness to the right kidney cortex is operator- and device- dependent. Moreover, the APV registry reflects only children and adolescents with overweight and obesity seen at specialized pediatric centers.

## Conclusions

We observed that extreme obesity, male gender and adolescent age are strongly correlated with increased liver echogenicity and elevated liver enzymes. Furthermore, we found a positive association between arterial hypertension and increased liver enzymes > 25 U/L, as well as between impaired glucose metabolism and elevated liver enzymes > 50 U/L. NAFLD is an early mediator reflecting metabolic disturbance, which should be detected to avoid further deterioration. Therefore, basic liver diagnostic evaluation for NAFLD including liver sonography and analysis of serum liver enzymes should be performed in all obese children with obesity. As first line therapy, lifestyle modification is recommended to avoid obesity related comorbidities.

## Data Availability

The datasets used and/or analyzed during the current study are available in anonymized and aggregated form from the corresponding author on reasonable request.

## Abbreviations

ALT: Alanine-Aminotransferase
APV: Adipositas-Patienten-Verlaufsdokumentation
AST: Aspartat-Aminotransferase
BMI: Body Mass Index
BP: Blood Pressure
DBP: Diastolic Blood Pressure
DHT: Dihydrotestosterone
gammaGT: gamma Glutamyltransferase
HbA1C: Glycated Haemoglobin
HDL-C: High Density Lipoprotein Cholesterol
HFF: Hepatic Fat Fraction
HMW: High-Molecur Weight
HOMA-IR: HomeOstasis Model Assessment-Insulin Resistance
LDL-C: Low-Density Lipoprotein Cholesterol
NAFLD: Non-Alcoholic Fatty Liver Disease
NASH: Non-Alcoholic Steatosis Hepatis
OGTT: Oral Glucose Tolerance Test
OR: Odds Ratio
S.D.: Standard Deviation
SBP: Systolic Blood Pressure
TC: Total Cholesterol
TG: Triglycerides
